# Outcomes of two different unbalanced segregations from a maternal t(4;10)(q33;p15.1) translocation

**DOI:** 10.1186/s12920-023-01491-1

**Published:** 2023-03-29

**Authors:** Judith Fan, T. Niroshini Senaratne, Jason Y. Liu, Michelle Bina, Julian A. Martinez-Agosto, Fabiola Quintero-Rivera, Jessica J. Wang

**Affiliations:** 1 Department of Medicine, University of California, 10833 Le Conte Avenue, Los Angeles, CA 90095 USA; 2 Department of Pathology and Laboratory Medicine, UCLA Clinical Genomics Center, University of California, Los Angeles, USA; 3 Department of Human Genetics, Sarah Lawrence College, Bronxville, USA; 4 Departments of Human Genetics, Pediatrics and Psychiatry, University of California, Los Angeles, USA

**Keywords:** 4q translocation, 10p translocation, Unbalanced translocation, Monosomy 4q, Monosomy 10p, Trisomy 4q, Trisomy 10p, Case report

## Abstract

**Background:**

Unbalanced translocations can cause developmental delay (DD), intellectual disability (ID), growth problems, dysmorphic features, and congenital anomalies. They may arise de novo or may be inherited from a parent carrying a balanced rearrangement. It is estimated that 1/500 people is a balanced translocation carrier. The outcomes of different chromosomal rearrangements have the potential to reveal the functional consequences of partial trisomy or partial monosomy and can help guide genetic counseling for balanced carriers, and other young patients diagnosed with similar imbalances.

**Methods:**

We performed clinical phenotyping and cytogenetic analyses of two siblings with a history of developmental delay (DD), intellectual disability (ID) and dysmorphic features.

**Results:**

The proband, a 38-year-old female, has a history of short stature, dysmorphic features and aortic coarctation. She underwent chromosomal microarray analysis, which identified partial monosomy of 4q and partial trisomy of 10p. Her brother, a 37-year-old male, has a history of more severe DD, behavioral problems, dysmorphic features, and congenital anomalies. Subsequently, karyotype confirmed two different unbalanced translocations in the siblings: 46,XX,der(4)t(4;10)(q33;p15.1) and 46,XY,der(10)t(4;10)(q33;p15.1), respectively. These chromosomal rearrangements represent two possible outcomes from a parent who is a carrier for a balanced translocation 46,XX,t(4;10)(q33;p15.1).

**Conclusion:**

To our knowledge, this 4q and 10p translocation has not been described in literature. In this report we compare clinical features due to the composite effects of partial monosomy 4q with partial trisomy 10p and partial trisomy 4q with partial monosomy 10p. These findings speak to the relevance of old and new genomic testing, the viability of these segregation outcomes, and need for genetic counseling.

**Supplementary Information:**

The online version contains supplementary material available at 10.1186/s12920-023-01491-1.

## Background

Unbalanced translocations can cause developmental delay (DD), intellectual disability (ID), growth problems, dysmorphic features, and congenital anomalies. They may arise de novo or may be inherited from a parent carrying a balanced rearrangement. It is estimated that 1/500 people is a balanced translocation carrier. The chance of a balanced translocation carrier having a live-born child with an abnormality varies by the specific chromosomal regions affected, but most risk figures range from 0 to 30% [1]. Balanced translocation carriers are typically asymptomatic but have a chance of having offspring with DDs and/or birth defects and an increased risk for miscarriage due to unbalanced segregation [1]. The outcomes of different chromosomal rearrangements have the potential to reveal the functional consequences of partial trisomy or partial monosomy.

The 4q deletion syndrome is a recurrent abnormality with an estimated incidence of 1:100,000 [2]. Features include DD, ID, growth failure, autism spectrum disorders, attention deficit disorders, as well as craniofacial, skeletal, digital, and cardiac anomalies [3–8]. On the other hand, duplications of 4q33-4q34 are associated with DD, mild-to-severe ID, growth delay, microcephaly, dysmorphic features, and digital anomalies [9]. Less common features include cardiac malformations, renal anomalies, cryptorchidism, umbilical hernia, and epilepsy. Deletions of 10p15.3 are associated with severe ID, DDs, craniofacial dysmorphism, hypotonia, brain anomalies, seizures, language impairment, and autistic behavior [10, 11]. Duplication of 10p15 has rarely been described in the literature.

The aim of this report is to present two siblings with a history of DD, ID and dysmorphic features, whose G-banded karyotype confirmed different reciprocal unbalanced translocations. These chromosomal rearrangements represent two possible outcomes from a parent who is a carrier of a balanced translocation. To our knowledge, this 4q and 10p translocation has not been described in literature. These findings speak to the viability of these segregation outcomes and can help guide genetic counseling for balanced carriers, and other young patients diagnosed with similar imbalances.

## Methods

### Participant characteristics

Two siblings with a history of DD, ID and dysmorphic features present for genetic counseling and evaluation. Parents are South Asian and non-consanguineous. Their first-born child is a phenotypically normal female with a reportedly normal karyotype and two healthy children. There was no family history of spontaneous abortions.

### Chromosomal microarray analysis (CMA)

CMA was performed on peripheral blood for patient 1 at the UCLA Clinical Microarray Laboratory using clinically validated protocols. A whole genome Single Nucleotide Polymorphism (SNP) oligonucleotide array was used to assess for imbalances in the genomic DNA sample tested. The assay compared patient 1’s DNA to an internal reference and to an external reference from 380 normal controls using the Affymetrix Genome-Wide SNP Array CytoScan™ HD (ThermoFisher Scientific). This array platform contains 2.6 million markers for Copy Number Variant detection, of which 750,000 are genotype SNPs and 1.9 million are non–polymorphic probes, for whole genome coverage. The analysis was performed using the Affymetrix Chromosome Analysis Suite (ChAS) software, version 3.1.0.15 (r9069).

### G-banded karyotype and fluorescence in situ hybridization (FISH)

Standard G-banded high-resolution chromosome analysis and metaphase FISH were performed for both patients and their mother at the UCLA Cytogenetics Laboratory following clinically validated protocols. Stimulated peripheral blood cultures were used. Giemsa-banded metaphase cells were analyzed and described according to ISCN 2016. Fluorescence in situ hybridization (FISH) was performed using the Vysis ToTelVysion Probe kit (Abbott Molecular) with probe cocktails specific to chromosomes 4p/q and 10p/q.

## Results

Patient 1, a 38-year-old female, was born via vaginal delivery at 36 weeks gestation to a 35-year-old mother and a 42-year-old father. Her birth was complicated by umbilical rupture and bleeding. She had motor and speech delays, attributed to possible anoxic injury at birth. During pre-operative evaluation for dental surgery at the age of 37, electrocardiogram (ECG) showed a low atrial rhythm (negative P waves) and slight slurring of the QRS, consistent with Wolff-Parkinson-White pattern. Transthoracic echocardiogram showed mild coarctation of the aorta. At age 38, she has mild ID, takes the bus independently to volunteer, and attends a two-year community college part-time. Other diagnoses include iron deficiency, migraine without aura, and generalized anxiety disorder. Height is 155 cm (10% percentile). She has hypotelorism, palpebral fissure length 3 cm, mild proptosis, low set ears, midface hypoplasia, thin upper lip, micrognathia, dental crowding with orthodontics, high arched palate, fifth digit brachydactyly, soft tissue syndactyly of the 2–4 toes, levoscoliosis, right knee hyperreflexia, and multiple small nevi.

Patient 2, a 37-year-old male, is the brother of patient 1. He has a history of DD and behavioral difficulties. At 8 months of age he could not sit and started physical therapy. He sat and walked at 3 years. First words were at 4 years of age. He was in special education but mainstreamed by the fourth grade. He had trouble speaking full sentences and received speech and occupational therapy. He was diagnosed with unilateral cryptorchidism at 3 years of age, which was successfully treated with hormone therapy. He exhibits aggressive behavior, treated with quetiapine. At age 37, he attends a day program. On echocardiogram he has a mildly thickened but functionally trileaflet aortic valve that is not clinically significant. He has hirsutism, brachycephaly, triangular head, metopic ridge, synophrys, broad nasal bridge, mild proptosis, ptosis, low set posteriorly rotated ears, micrognathia, short neck with sloping shoulders, pectus excavatum, mild fifth finger clinodactyly, soft tissue syndactyly of the toes, and levoscoliosis.

**Fig. 1:**
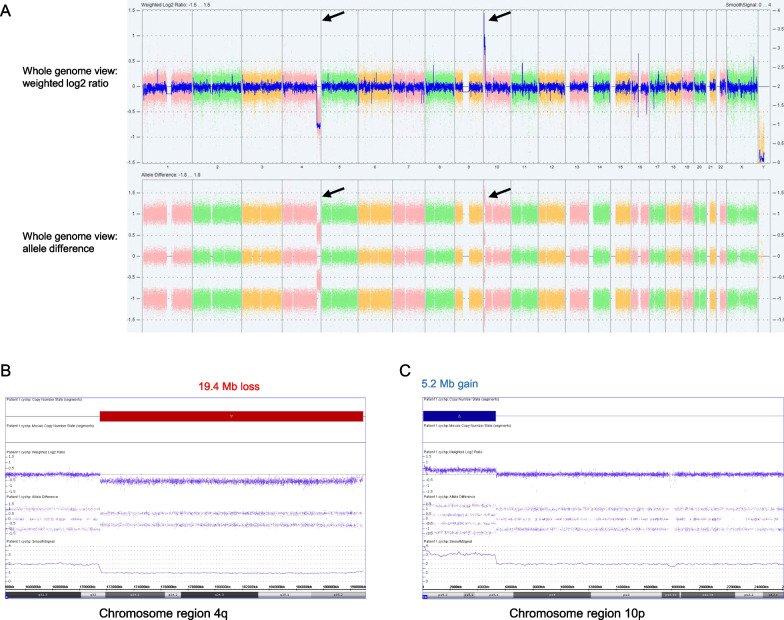
Chromosomal microarray analysis (CMA) for Patient 1. **A** Whole genome view of CMA data, showing weighted log2 ratio (top panel) and allele difference (bottom panel). These data show a copy number loss on chromosome 4, with a corresponding decrease in allele difference, and a copy number gain on chromosome 10, with a corresponding increase in allele difference. **B** View of CMA data for the chromosome 4q region, highlighting log2 ration -0.5, decrease in allele difference (two tracks, normal pattern is 3 tracks) and smooth signal of 1 all representing a 19.4 Mb monoallelic terminal loss (red bar). **C** View of CMA data for the chromosome 10p region, highlighting log2 ration 0.5, increase in allele difference (four tracks tracks) and smooth signal of 3 all representing a 5.2 Mb terminal gain (blue bar). Screenshots were taken from the Chromosome Analysis Suite (ChAS) software from Affymetrix, Inc

Patient 1’s CMA showed a loss minimally spanning 19.4 Mb of 4q33→4q35.2 and a gain minimally spanning 5.2 Mb of 10p15.3→10p15.1 (arr[GRCh37] 4q33q35.2(171509635_190957473) × 1,10p15.3p15.1(100026_5313662) × 3) (Fig. 1). The loss on 4q involves 96 curated RefSeq genes, while the gain on 10p involves 38 curated RefSeq genes (gene content is provided in Additional file 1: Table S1). These findings indicate a terminal loss from the long arm of chromosome 4 and a terminal gain involving the short arm of chromosome 10. No additional copy number changes of known clinical significance or long contiguous stretches of homozygosity were identified.

**Fig. 2:**
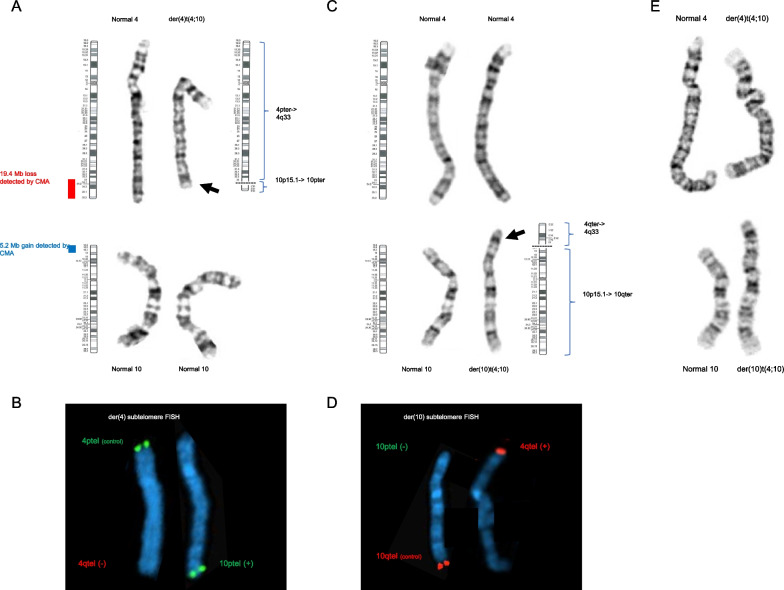
Chromosome analysis and fluorescence in situ hybridization (FISH) for Patient 1 and 2. **A** G-band chromosome analysis for Patient 1 showed one normal copy of chromosome 4, a derivative chromosome 4, and two normal copies of chromosome 10. Standard ideograms are shown next to the normal chromosomes, as well as a hypothetical ideogram for the derivative chromosome 4. The red and blue bars highlight the regions that were found by CMA to be lost/gained. **B** Metaphase FISH for Patient 1, focusing on the derivative chromosome 4. This chromosome showed a 4p-specific signal but was missing a 4q-specific signal (left). In a separate hybridization, this chromosome was found to hybridize with a 10p-specific FISH probe (right). **C** Chromosome analysis for Patient 2 showed two normal copies of chromosome 4, one normal copy of chromosome 10, and a derivative chromosome 10. Standard ideograms are shown next to the normal chromosomes, as well as a hypothetical ideogram for the derivative chromosome 10. **D** Metaphase FISH for Patient 2, focusing on the derivative chromosome 10. This chromosome showed a 10q-specific signal but was missing a 10p-specific signal (left). In a separate hybridization, this chromosome was also found to hybridize with a 4q-specific FISH probe (right). **E** Chromosome 4 and 10 karyotype of the siblings’ mother

Standard chromosome analysis identified an abnormal karyotype, with a derivative chromosome 4 resulting from an unbalanced (4;10) translocation, with breakpoints consistent with those observed by CMA: 46,XX,der(4)t(4;10)(q33;p15.1) (Fig. 2A). Metaphase FISH with subtelomere probes targeting chromosomes 4 and 10 confirmed the unbalanced translocation, with loss of a 4q-specific signal and gain of a 10p-specific signal: ish der(4)t(4;10)(q35.2−,p15.3+)(D4S2930-,10pTEL006+) (Fig. 2B).

Patient 2 underwent G-banded karyotype and metaphase FISH. These studies found that he also carried an unbalanced (4;10) translocation but with a derivative chromosome 10: 46,XY,der(10)t(4;10)(q33;p15.1) (Fig. 2C). Metaphase FISH confirmed loss of a 10p-specific signal and gain of a 4q-specific signal: ish der(10)t(4;10)(q35.2+,p15.3−)(D4S2930+,10pTEL006−) (Fig. 2D).

Parental studies showed that mother carries a balanced translocation, 46,XX,t(4;10)(q33;p15.1) (Fig. 2E). The father’s karyotype was normal.

## Discussion

This report highlights different features associated with two different outcomes of meiotic segregation in a balanced translocation carrier. The mother of our proband was found to carry a balanced (4;10) translocation. The daughter, patient 1, inherited the derivative chromosome 4, resulting in partial monosomy 4q (loss of 19.4 Mb) and partial trisomy 10p (gain of 5.2 Mb), with breakpoints defined by CMA. The son, patient 2, inherited the derivative chromosome 10, resulting in partial trisomy 4q and partial monosomy 10p. While patient 2 did not get a CMA, the breakpoints of his translocation can be inferred based on those identified in his sister, suggesting a 19.4 Mb gain of 4q and a 5.2 Mb loss of 10p. Together, these patients help expand the phenotypic spectrum associated with copy number variation involving 4q and 10p and identify possible candidate genes associated with these phenotypes. The clinical data for our patients compared to individuals reported in the literature and analyzed by CMA is summarized in Table 1.

**Table 1:** Comparison with other reported cases with similar genomic imbalances

Phenotype	Patient 1	Patient 2	Strehle et al. [2]	Strehle et al. [2]	Xu et al. [8]	Thapa et al. [9]
Study description	4q33q35.2 deletion; 10p15.3p15.1 duplication	4q33q35.2 duplication; 10p15.3p15.1 deletion	Case series of 3 patients with 4q-syndrome	Review of 20 patients with deletions ranging from 160 kb to 25.7 Mb; two individuals with an additional copy number variant	1.6 Mb deletion of 4q32.3q34.3	Distal 4q duplication
No. of patients in study			3	20	1	2
Karyotype/coordinates	arr[hg19] 4q33q35.2(171,509,635-190,957,473) × 1,10p15.3p15.1(100,026-5,331,662) × 3 46,XX,der(4)t(4;10)(q33p15.1)	46,XY,der(10)t(4;10)(q33;p15.1)	Patient 1: 46,XX,del(4)(q33).ish del(4)(q33)(RP11-140M23-,DJ963K6-)dn Patient 2: 46,XY.arr cgh 4q31.3q35.2(153,114,490-191,034,023) × 1 dn and 46,XY.arr cgh 6p25.3(103,562-1,559,010) × 3 dn build not indicated Patient 3: 46,XX.arr cgh 4q22.1q23(93,972,073-101,811,206) × 1 dn	Mixed	arr[hg18] 4q32.3q34.3(167,236,114-178,816,031) × 1	Mixed
Developmental delay	1	1	1	18	1	2
Hypotonia			1	10		
Intellectual disability^a^	1	1	0^b^	10^b^	0^b^	2
Craniofacial findings						
Cleft lip/palate			2	6		
Macrocephaly				4		
Microcephaly			1	4		
Hypotelorism	1			1		
Hypertelorism			1	4		1
Ear anomalies		1	2	11	1	
Frontal bossing/high forehead/prominent forehead			1	4		2
Broad nasal bridge		1	2	3		1
Mouth/lip dysmorphology	1		2	4		
Micrognathia/retrognathia/chin dysmorphology	1	1	3	8		1
Dental findings	1			7		
High arched palate 1			1			3
Philtrum abnormality				6		
Nose dysmorphism				12		
Skeletal findings						
Growth deficiency/short stature		1	1	10	1	
Scoliosis	1	1		2		
Brachydactyly/small hands and/or feet 1			4		1	
Clinodactyly/overlapping/malpositioned fingers/toes		1	2	11	1	
Skin findings						
Nevi, hyperpigmentation, ichthyosis, etc	1			5		
Syndactyly, soft tissue of fingers/toes	1	1		1		
Cardiovascular findings	1		2	9	1	
Immunological symptoms						
Asthma				3		
Recurrent infections				2		
Neurological findings						
Brain MRI/CT findings^d^			1	4		
Seizures				5		
Genitourinary	1	1	1	9	1	1
Endocrine	1			3		
Ears, nose, throat						
Hearing impairment				3		

Phenotype	DeScipio et al. [11]^c^	Lindstrand et al. [10]	Kohannim et al. [21]	Cingoz et al. [3]	Shah et al. [23]
Study description	Review of 19 unrelated individuals with submicroscopic 10p15.3 deletions. Clinical history available for 12 out of 19 patients, with deletions ranging in size from 154 to 3706 kb	4 Patients with partial overlapping 10p deletions	Trisomy 10p11.22p15.3 and monosomy 7p22.3	4q35 deletion and 10p15 duplication in two family members	Case report of 4q34.3q35.2 11.5 Mb deletion and 10p15.3p12.1 29.3 Mb duplication
No. of patients in study	19	4	1	2	1
Karyotype/coordinates	Patient 1: arr[hg18] 4q32.1q35.2(157,561,683-191,133,858) × 3 Patient 2: arr[hg18] 4q32.2q34.3(163,694,953-179,222,949) × 3	Patient 1: 46,XY,t(1;10;5)(q32;p12;q31) Chr1: 195,145,260-195,382,317 Chr10: 4,592,014-4,883,438 Chr10: 19,135,572-19,639,499 Chr10: 20,747,991-20,909,373 Chr5: 124,638,176-125,261,854 Patient 2: Chr10: 32,427,01-34,62,269 Patient 3: Chr10: 11,974,466-12,211,025 Patient 4: 45,XX,der(10;15)(10qter→10p15::15p11)dn hg18	46,XY,der(7)t(7;10)(p22.3;p11.22).arr 7p22.3(0-749,854) × 1,10 p15.3p11.22 (0-33,408,955) × 3,10p11.22(33,039,622-33,408,955)x4dn	[ish cgh del(4)(q35qter)] and [ish cgh dup(10)(p15ter)]	46,XY,der(4)t(4;10)(q34.3;p12.1)dn.arr [hg19] 4q34.3q35.2(179,589,516-191,154,276) × 1,10p15.3p12.1(0-29,304,999) × 3
Developmental delay	17	4	1		1
Hypotonia	9	2			1
Intellectual disability^a^	7	4	0^b^	2	1
Craniofacial findings					
Cleft lip/palate		1			
Macrocephaly	4				
Microcephaly	1	1			
Hypotelorism				2	
Hypertelorism	2	2	1		
Ear anomalies	4	4	1	2	
Frontal bossing/high forehead/prominent forehead	5	1	1		
Broad nasal bridge	2	1			
Mouth/lip dysmorphology	6	1			
Micrognathia/retrognathia/chin dysmorphology	2	1	1		1
Dental findings	1				1
High arched palate 1	1	1			
Philtrum abnormality	2	1			
Nose dysmorphism	2	2		1	
Skeletal findings					
Growth deficiency/short stature	6	3			
Scoliosis	1	1			1
Brachydactyly/small hands and/or feet 1					
Clinodactyly/overlapping/malpositioned fingers/toes	3	1			
Skin findings					
Nevi, hyperpigmentation, ichthyosis, etc	1				
Syndactyly, soft tissue of fingers/toes	1				
Cardiovascular findings	3			1	1
Immunological symptoms					
Asthma	1		1	2	
Recurrent infections	2	2	1	1	
Neurological findings					
Brain MRI/CT findings^d^	4	3			
Seizures	3	2			
Genitourinary	4	3		1	
Endocrine					
Ears, nose, throat					
Hearing impairment		3			

Empty cells = not reported

^a^Including learning disability but not ‘mild’ learning disability

^b^Includes children < 5 years old and/or termination

^c^Only 12 out of 19 patients had detailed phenotypic information

^d^Including findings such as hydrocephalus, possible absence of corpus callosum

Regions 4q32.3–q34.3 and 4q33–q34 have been implicated as critical regions accounting for 4q deletion and duplication phenotypes, respectively [8, 9]. Xu et al. reported an 8-month-old male with growth delay, skeletal anomalies, ventricular septal defect, secundum atrial septal defect thickened dysplastic pulmonary valve with stenosis and regurgitation, and a de novo 11.6 Mb deletion of 4q32.3q34.3 [8]. The authors concluded that the region spanning *TLL1* (MIM# 606742), *HPGD* (MIM# 601688), and *HAND2* (MIM# 602407) is critical for the development of congenital heart defects in the 4q deletion syndrome (Additional file 1: Table S2). The overlap between their patient’s deletion and that seen in our Patient 1 is approximately 7.0 Mb and involves 31 curated RefSeq genes, including *HGPD* and *HAND2* but not *TLL1. HGPD* encodes 15-hydroxyprostaglandin dehydrogenase and may play a role in remodeling of the ductus arteriosus [12]. *HAND2,* heart and neural crest derivatives expressed 2, encodes a helix-loop-helix transcription factor that regulates several cell types during embryonic development [13]. Deletions of *HAND2* have been associated with congenital heart defects [2].

Regarding the 4q duplication phenotypes, Thapa et al. proposed 4q33–q34 to be a critical region and suggested that dosage sensitivity of one or both of *GLRA3* (MIM# 600421) and *GPM6A* (MIM# 601275) may be associated with DD and ID, while *HAND2* may be critical for craniofacial development [9]. The minimal region of overlap between the two patients reported by Thapa et al. and the predicted gain in our Patient 2 is approximately 7.5 Mb and involves 33 curated RefSeq genes, including *GLRA3*, *GPM6A*, and *HAND2*. *GLRA3* encodes the alpha-3 subunit of the neuronal glycine receptor essential for synaptogenesis while *GPM6A* [14]. *GPM6A* is a neuronal transmembrane protein that is thought to play a role in neurite growth, neuronal differentiation, and synapse formation. Gregor et al. reported a *GPM6A* duplication in an individual with learning disability, behavioral problems, and minor facial dysmorphism and studied the impact of dosage alterations of *GPM6A* on *Drosophila melanogaster* [15]. Their findings implicated the overexpression of *GPM6A* in the cognitive phenotype seen in their patient.

A 1.6 Mb region in 10p15.3 has been proposed as a critical region for ID and speech impairment, with two proposed candidate genes, *UCN3* (MIM# 605901) and *IL15RA* (MIM# 601070) [10]. The region of overlap between the proposed 1.6 Mb critical region and the region deleted in our patient is approximately 622 kb but does not include either *UCN3* or *IL15RA*. DeScipio et al., documented 19 individuals with submicroscopic deletions of 10p15.3 and found that *ZMYND11* (MIM# 608668) was deleted in all individuals but one and *DIP2C* (MIM# 611380) was deleted in all but one other individual. They suggested that haploinsufficiency of these genes contributes to the features of the 10p15 deletion phenotype [11]. *ZMYND11* has been associated with autosomal dominant ID and both *ZMYND11* and *DIP2C* are expressed in the brain [16]. Both genes were deleted in our patient 2.

Stone et al. reported siblings with a paternally inherited derivative chromosome 9 resulting in a 10p14p15 duplication [17]. Features in this family included dysmorphic features, decreased IgG levels, mild learning problems and eye anomalies. Benzacken et al. described a patient with a de novo 10p14pter duplication presenting with significant hypotonia, DD, agenesis of the corpus callosum, glottic stenosis, and dysmorphic features [18]. In contrast, duplication involving the entire short arm of chromosome 10 has been well described and is associated with dysmorphic features, DD, ID, growth retardation, hypotonia, high-arched/ cleft palate, organ malformations, skeletal abnormalities, and clubfoot [19–21].

Translocations between 4q and 10p have previously been described in association with multiple congenital anomalies, but contain different breakpoints than our patients’. One report documented an unbalanced translocation between 4q35 and 10p11.23 resulting in a duplication of 10p11.23pter found in a child with severe failure to thrive, moderate delays, multiple congenital anomalies, facial dysmorphism, and craniosynostosis [22]. Another report identified a cryptic reciprocal translocation resulting in a 4q35 deletion and 10p15 duplication in proband and her maternal aunt who both had ID, dysmorphic features, and immunological symptoms [3]. Shah et al. reported a 4q34.3q35.2 11.5 Mb deletion and 10p15.3p12.1 29.3 Mb duplication in a patient with global DD, severe ID, Marfanoid phenotype, moderate aortic regurgitation due to progressive aortic root dilatation, and a “string of beads” appearance of the femoral artery typical of medial fibroplasia type of fibromuscular dysplasia [23]. The deletion of 4q reported in this patient was slightly larger but overlapping that seen in our patient 1 (breakpoints within 2 Mb of each other), while the 10p duplication was significantly larger (29.3 Mb versus 5.2 Mb).

**Fig. 3:**
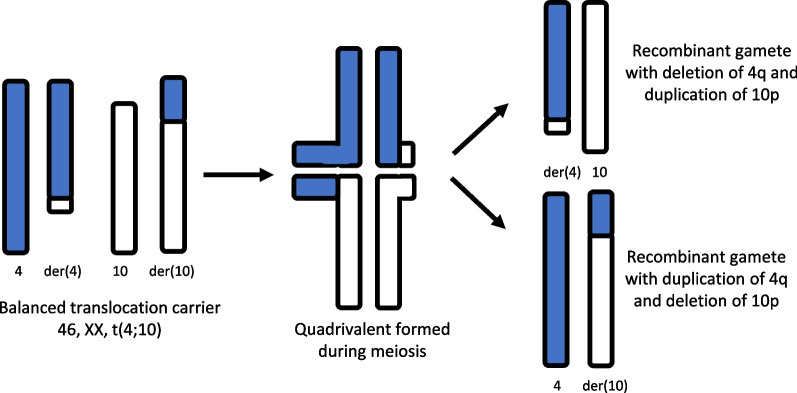
Diagram depicting meiosis of a balanced translocation carrier resulting in reciprocal unbalanced translocations in the offspring. The balanced translocation chromosomes are paired, forming quadrivalent, then segregated during the meiosis I, into two different unbalanced translocations arrangements

Finally, these cases illustrate how balanced translocation carriers gives rise to unbalanced translocations in offspring, specifically, how quadrivalent chromosomes of a balanced translocation carrier during meiosis give rise to reciprocal unbalanced translocations (Fig. 3). Accurate and comprehensive family history information assists in diagnosis and tracking of genetic conditions throughout a family. Balanced carriers of chromosomal rearrangements can have reproductive histories that include infertility, recurrent pregnancy loss, fetal anomalies, and offspring with abnormalities. Therefore, collection of a targeted family history can identify individuals with increased risks to carry a chromosomal rearrangement based on their own reproductive history or the history of close relatives. Identification and evaluation of at-risk relatives allows for preconception consideration of various reproductive options, including the use of assistive reproductive technology, and the prevention of added negative psychological stress due to learning of an increased risk for abnormalities during an ongoing pregnancy.

## Conclusions

To our knowledge, this is the first report of siblings with reciprocal unbalanced translocations between 4q33q35.2 and 10p15.3p15.1. Both have a history of DD, learning problems, and characteristic craniofacial features, with varying degrees of special needs. These findings speak to the viability of these segregation outcomes, their natural history and need of further characterization of CMA findings, and for genetic counseling of carriers of this translocation. Moreover, we compared clinical features due to the composite effects of unbalanced translocations. The outcomes of different chromosomal rearrangements have the potential to reveal the functional consequences of specific partial trisomy or partial monosomy.

## Supplementary Information

**Additional file 1. Table S1.** Lists of genes contained within the genomic imbalances. **Table S2.** Genes that may contribute to the patients’ phenotypes.

## Data Availability

The study data has been deposited and made publicly available through dbVar at accession number nstd228 [24].

## Abbreviations

DD: Developmental delay
ID: Intellectual disability
CMA: Chromosomal microarray analysis
ECG: Electrocardiogram
SNP: Single nucleotide polymorphism
FISH: Fluorescence in situ hybridization
